# Use of corticosteroids in COVID-19 patients vs. acute respiratory distress syndrome of other etiologies: Are there any differences?

**DOI:** 10.5339/qmj.2021.30

**Published:** 2021-09-16

**Authors:** Pedro Barrera-López, Erika D. Pérez-Riveros, Mariana Vásquez Roldan, Silvia Marcela Ballesteros, José A. De la Hoz-Valle, José Moreno-Montoya

**Affiliations:** ^1^Clinical Studies and Clinical Epidemiology Division, Fundación Santa Fe de Bogotá, Bogotá, Colombia E-mail: pbarreramed@gmail.com

## Introduction

The disease resulting from infection by severe acute respiratory syndrome coronavirus 2 (SARS-CoV-2), widely known as coronavirus disease 2019 (COVID-19), has been classified as a global pandemic. It is characterized by respiratory compromise, which may include multisystemic involvement due to endothelial damage as well as activation of multiple thromboinflammatory mechanisms,^1,2^ leading to various serious clinical stages such as multiple organ failure and death due to Acute Respiratory Distress Syndrome (ARDS).^1^ In addition, the increased demand for health services, including the need for intensive care units and advanced vital resources such as mechanical ventilation or extracorporeal membrane oxygenation and trained personnel, poses serious challenges in choosing a suitable treatment.^3,4^

In the absence of a standard treatment, the clinical approach to those affected has focused on pharmacological management, particularly the use of antivirals and immunomodulators.^5^ According to the phases of the immunological response,^6^ the use of glucocorticoids has become widespread given its effect on the control of the inflammatory cascade from its origin (blockade of phospholipase A2) to its wide pharmacological availability and low cost. To date, the results have been controversial. At the beginning of the pandemic, these drugs were not recommended for use in COVID-19 patients.^7^ Nonetheless, current preliminary results of a clinical trial in the RECOVERY group show a decrease in mortality in patients receiving dexamethasone,^8^ raising the possibility of the systematic use of glucocorticoids for COVID-19 disease.^9^

The purpose of this review is to perform a rapid assessment of the evidence for and against the use of glucocorticoids to prevent progression to ARDS and related mortality in both COVID-19 and ARDS mediated by other pathologies.

## Methods

**Figure 1.**
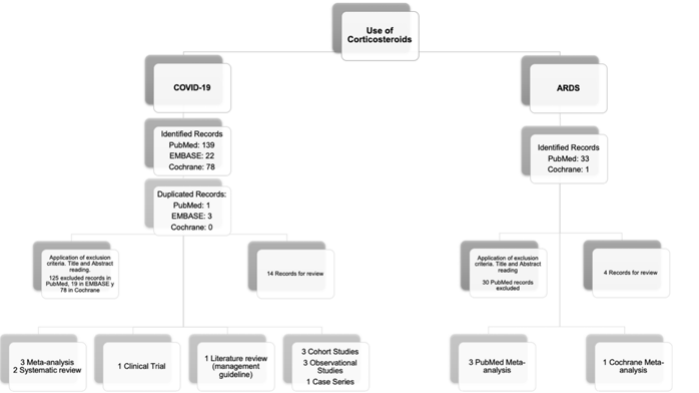


A systematic review was carried out in the “PubMed,” “EMBASE,” and “Cochrane” databases with the following MESH terms: Articles published in the English and Spanish languages were included. For articles related to COVID-19, the deadline was July 31, 2020, and the start date was December 2019. For the meta-analysis related to ARDS from other causes, the start date was 2017, and the search and review time was two months. To assess the effect on COVID-19 patients, only original studies and meta-analyses published to date for other ARDS etiologies were included. Each search yielded 235 and 34 publications, respectively. Studies with samples smaller than ten patients; studies that evaluated the use of glucocorticoids in patients with rheumatological disease, autoimmune disease, and transplant or neoplasm recipients; reviews focused on neonates; lung maturation studies; and one meta-analysis on ARDS and septic shock were excluded. Finally, 14 original articles and 4 meta-analyses were obtained for review (Figure 1).

## Results

The main findings are summarized in the table. In general, a comparative analysis of the findings on the use of glucocorticoids for the treatment of COVID-19 and other causes of ARDS revealed great heterogeneity among treatment types using prednisone, methylprednisolone, dexamethasone, and hydrocortisone. Treatment schedules ranged from low doses to pulses of steroids in both groups, and for COVID-19, dexamethasone and methylprednisolone were used more frequently than for ARDS of other causes, where the most used were methylprednisolone and dexamethasone. Only the RECOVERY study specified the application protocol (table 1). Furthermore, it was evidenced that the effectiveness in reducing mortality, recovery from ARDS, or reduction in hospitalization time remained inconclusive, with high heterogeneity in the findings. Two observational studies in China (1326 patients),^10,11^ described a greater use of glucocorticoids in seriously ill patients with an increase in mortality; even a meta-analysis by Lu et al. (May, 2020) and the WHO provisional guidelines of January 28, 2020 advised against its use.^12^

## Discussion

Despite the heterogeneity in the findings described above, an understanding of the pathophysiology and evolution of COVID-19 disease, mainly the syndrome of innate release of cytokines mediated by the immune system (macrophages and proinflammatory granulocytes) through the production of TNF-α and IL-6 was achieved^6^; in addition to recognition of the phases of SARS-CoV-2 infection, which initiates a response mainly mediated by “natural killer” cells and cytotoxic T lymphocytes to the secretion of type I interferon by infected cells.^5^ Subsequently, a series of deleterious mechanisms are activated, leading to lung tissue infiltration and damage progressing to ARDS, and endothelial impairment. With the foregoing, the use of corticosteroids alone or concomitantly with other immunomodulatory therapies for the management of severe forms of COVID-19 has been reconsidered and recommended, to reduce mortality in patients who have already developed lung damage.^13^

The most recent systematic reviews and meta-analyses (Table 1), besides corroborating previous findings, demonstrate a decrease in the duration of mechanical ventilation compared with the usual management, without evidence of a significant increase in side effects. Only lower clearance of the virus was observed, but low certainty in the findings remained, given the heterogeneity of the studies.

In other ARDS etiologies, marginal benefits of drug therapies have been shown over time. In the four meta-analyses that condensed 17 clinical trials and more than 1,500 patients, the results are equally heterogeneous; cases of reduction in mortality or duration of mechanical ventilation are specific cases that do not allow recommendations to be made for systematic application. In one of the meta-analyses, there is even evidence of a decrease in mortality at day 28 for all patients,^14^ a finding that so far is the main argument for the use of dexamethasone in COVID-19^8^; however, this finding it is not demonstrated in other settings. The findings of side effects of steroids in ARDS are similar, mainly the risk of superinfection, which is not shown to be persistently increased in all patients.

Within the pathophysiology of COVID-19 disease, the progression to severe forms does not depend only on the infectious agent but also on the interaction with the host's immune system, in which the human leukocyte antigen (HLA) plays a central role. HLA represents one of the most polymorphic systems that participate in the immune response, and its various polymorphisms have been associated with worse outcomes both in COVID-19^6^ and in other viral infections, such as Influenza AH1N1.^15^ Therefore, we consider that glucocorticoid therapy for patients with COVID-19 should not be based solely on pulmonary involvement. Hence, the indication should be individualized according to the patient's immune response and immunophenotype, particularly in those with a proinflammatory state that can decline over the course of the disease, based on laboratory markers that have been associated with a worse outcome and a deleterious immune response, such as IL-6 and tumor necrosis factor alpha.

## Conclusion

It is hypothesized that the demonstrated benefits of steroids in modulating the inflammatory response, slowing the progression to ARDS and associated mortality, both for COVID-19 and for ARDS due to other causes, will be enhanced if the type of immune response can be identified. Administration of a treatment aimed at the specific pathophysiological mechanism will also allow reduction of the possible adverse results associated with the use of these drugs. We understand that the limited access and costs associated with the collection and processing of immunological profiles or HLA subtypes compared with the benefits of the application of glucocorticoids limit the possibility of carrying out a targeted treatment according to the immunophenotype, but we believe that this is as area of potential research and development to optimize individualized therapies.

## Limitations

The main limitations of the study were related to time, phrases and languages that could be analyzed, which restricted the criteria for article inclusion in the meta-analysis, because there are many other references of great importance in languages such as Italian and Mandarin. Likewise, the time interval covered by the review is an important limitation because the production time in COVID-19 is in constant growth, and it is not possible to keep reviews like this one completely up-to-date. It is important to note that the objective was always to carry out a systematic review of the literature with a subjective comparison and analysis, and the application of a statistical analysis (meta-analysis) was not within the objectives and design of the study.

## Addendum

After the search window for inclusion of articles in the systematic review, a meta-analysis published on September 2, 2020 was found that evaluated the usefulness of steroids to reduce mortality in COVID-19. This included seven studies with 1703 patients, of which 678 patients received dexamethasone, hydrocortisone, or methylprednisolone, and 1025 patients received a placebo. The conclusion is that patients who receive glucocorticoids systematically presented a reduction in mortality at day 28, compared with the placebo.^16^

In addition, on February 25, 2021, the RECOVERY group updated its results where the use of dexamethasone was associated with a lower mortality on day 28 among patients who required the use of invasive mechanical ventilation or supplemental oxygen, which was not the case for patients with noninvasive mechanical ventilation.^17^ These new results continue to reinforce our recommendation that the benefits of steroids can be enhanced by applying targeted therapy to specific immunophenotypes.

### Disclosure of interest

The authors have no potential competing interest to report.

### Funding information

The authors did not participate in any open calls, nor did they receive any financial support from their institution or any other entity during the development of the study. Therefore, they did not receive any research funding, nor are the currently receiving any research funding, the reason why there are no competing interests.

### Acknowledgments

Not applicable

## Figures and Tables

**Table 1:** 

Corticosteroid use in COVID-19 patients							

Type of Study	Title	Corresponding Author	Journal	DOI	Selection Criteria	Number of Cases	Used Drug

Meta-analysis	Effectiveness and safety of glucocorticoids to treat COVID-19: a rapid review and meta-analysis	Chenyan Zhou	Annals of Translational Medicine	10.21037/atm-20-3307	ACE - Cohort Study	23 Studies 1 ACE 22 Cohorts	For the COVID-19 studies, methylprednisolone was used

Meta-analysis	Antiviral agents, glucocorticoids, antibiotics, and intravenous immunoglobulin usage in 1142 patients with coronavirus disease 2019: a systematic review and meta-analysis	Wenfang Li	Polish Archives of Internal Medicine	10.20452/pamw.15543	ACE - Cohort Study	6 Studies 1 ACE 5 Observational	1 methylprednisolone study, the rest were unspecified

Meta-analysis	Drug treatments for covid-19: Living systematic review and network meta-analysis	Reed AC Siemieniuk	BMJ : British Medical Journal	10.1136/bmj.m2980	ACE	23 ACE's	methylprednisolone dexamethasone

Systematic review	Role of corticosteroid in the management of COVID-19: A systemic review and a Clinician's perspective	Awadhesh Kumar Singh	Diabetes & Metabolic Syndrome	10.1016/j.dsx.2020.06.054	ACE - Observational	1 ACE 5 Observational	1 methylprednisolone 1 dexamethasone (RECOVERY) Other unknown

Systematic review	Treatment options in people with COVID19: Selecting the best armamentarium against the novel virus	Deep Dutta	JPMA. The Journal of the Pakistan Medical Association.	10.5455/JPMA.22	Not specified	289 studies - does not specify which studies were for corticosteroids	methylprednisolone

Clinical Trial	Dexamethasone in Hospitalized Patients with Covid-19 – Preliminary Report	RECOVERY Collaborative Group	The New England Journal of Medicine	10.1056/NEJMoa2021436	Open ACE - inpatient	2104 Treatment Group 4321 Control Group	Oral or IV dexamethasone

Literature review	A critical evaluation of glucocorticoids in the management of severe COVID-19	Cinzia Solinas	Cytokine & Growth Factor Reviews.	10.1016/j.cytogfr.2020.06.012	Literature review	7 Treatment Guidelines	methylprednisolone dexamethasone

Cohort	Impact of glucocorticoid treatment in SARS-COV-2 infection mortality: A retrospective controlled cohort	Ana Fernández Cruz	Antimicrobial Agents and Chemotherapy.	10.1128/AAC.01168-20	According to the management received upon hospital admission	463 patients - 396 receive treatment 67 control group	methylprednisolone

Cohort	Historically controlled comparison of glucocorticoids with or without tocilizumab versus supportive care only in patients with COVID-19-associated cytokine storm syndrome: results of the CHIC study	Sofia Ramiro	Annals of the Rheumatic Diseases	10.1136/annrheumdis-2020-218479	Patients with cytokine release syndrome in COVID-19	86 Treatment Group and 86 Control Group	methylprednisolone (pulsed)

Cohort	Clinical efficacy of glucocorticoid on the treatment of patients with COVID-19 pneumonia: A single-center experience	Yan Hu	Biomeédecine & Pharmacotheérapie	10.1016/j.biopha.2020.110529	Presence of lung consolidations - COVID-19 pneumonia	308 patients	methylprednisolone (97.7%) prednisone (17.4%)

Observational	Eficacia de los pulsos de corticoides en pacientes con síndrome de liberación de citocinas inducido por infección por SARS-CoV-2	José Luis Callejas Rubio	Medicina Cliénica.	10.1016/j.medcli.2020.04.018	Patients with cytokine release syndrome in COVID-19	92 patients	methylprednisolone

Observational	Analysis of the clinical characteristics, drug treatments and prognoses of 136 patients with coronavirus disease 2019	Yonggang Chen	Journal of Clinical Pharmacy and Therapeutics.	10.1111/jcpt.13170	Not applicable	136 patients	methylprednisolone

Observational	Clinical outcomes of COVID-19 in Wuhan, China: A large cohort study	Jiao Liu	Annals of Intensive Care	10.1186/s13613-020-00706-3	Not applicable	1190 patients	

Case Series	Short-term dexamethasone in Sars-CoV-2 patients	Vijairam Selvaraj	Rhode Island Medical journal.	PMID: 32570995	Disease Severity(1980–2006)	21 patients	IV pexamethasone

Corticosteroids use in ARDS of other etiologies							

Type of Study	Title	Corresponding Author	Journal	DOI	Selection Criteria	Number of Cases	Used Drug

Meta-analysis	Do glucocorticoids decrease mortality in acute respiratory distress syndrome? A meta-analysis	Ritesh Agarwal	Respirology : Official Journal of the Asian Pacific Society of Respirology.	10.1111/j.1440-1843.2007.01060.x	ECAs (1980–2006)	6 ACEs - number of patients not specified	5 methylprednisolone 1 hydrocortisone

	Impact of corticosteroids on mortality in patients with acute respiratory distress syndrome: A systematic review and meta-analysis	Nobuyuki Horita	Internal Medicine	10.2169/internalmedicine.54.4015	Use of methylprednisolone for ARDS	4 ACEs 1 ACE - secondary analysis 6 cohorts 949 patients in total (461 treated–488 control)	methylprednisolone 3 high-dose studies 9 low-dose studies

Prolonged glucocorticoid treatment is associated with improved ARDS outcomes: analysis of individual patients' data from four randomized trials and trial-level meta-analysis of the updated literature	G. Umberto Meduri	Intensive Care Medicine	10.1007/s00134-015-4095-4	ACEs using methylprednisolone	Added 8 ACEs - 4 methylprednisolone and 4 with hydrocortisone	322 patients treated with methylprednisolone (118 early tratment < 72 h–204; initial treatment on days 5–7) 297 with hydrocortisone

Pharmacological agents for adults with acute respiratory distress syndrome	Sharon R Lewis	The Cochrane Database of Systematic Reviews.	10.1002/14651858.CD004477.pub3	ACE (2000-2018)	Steroids - 7 studies (643 patients)	hydrocortisone methylprednisolone budesonide


Abbreviations: ARDS = acute respiratory distress syndrome; COVID-19 = coronavirus disease 2019; SARS-CoV-2 = severe acute respiratory syndrome coronavirus 2; ACE =  ; CMV = ; WMD = ; CRP = C-reactive protein; OTI = ; SLC =

**Table 1:** 

Corticosteroid use in COVID-19 patients								

		Results			Treatment Complications			

Mortality	Hospital stay	Severity Progression	Signs and Symptoms	Other Findings	Reported	Explanation	Study Limitations	Conclusions

The drug does not reduce mortality (RR: 2.00, 95% CI 0.69	Prolonging WMD = 2.4 days, 95% CI = 1.4–3.4, I2 = 0.0% (5872 patients)	Duration of lung inflammation WMD = -1 day, C = I 95% CI: -2.91–0.91 (4709 patients)	Reduction in fever WMD = -3.23 days, 95% CI = -3.56 to -2.90	No significant findings for lung inflammation or mortality in SARS and MERS	Yes	Extension of hospital stay Risk of co-infections.	The analysis was not exclusive to COVID-19; studies of SARS and MERS were included Classification of the recommendations for COVID-19 as "VERY LOW" according to GRADE	They do not recommend the routine use of systemic glucocorticoids in COVID-19 patients

Increased risk: OR: 2.43; 95% CI = 1.44–4.10; *p* = 0.001; I2 = 61.9%	Not specified	Not specified	Not specified	No effect was found on mortality derived from the use of IVIg	No	No	Selection criteria for receiving therapies were not specified in retrospective studies. The glucocorticoid analysis only included observational studies with high heterogeneity.	Among the multiple treatment options, corticosteroids show a harmful effect in patients with COVID-19.

Reduces mortality: (RR: 0.88, 95% CI = 0.80–0.97) 37 fewer patients per 1000 (95% CI = 63–11, fewer per 1000 patients) (8654 patients).	Not analyzed for glucocorticoids	Reduces the need for CMV RR: 0.74 95% CI = 0.59–0.93 30 fewer patients per 1000 (95% CI = 48–8 fewer per 1000 patients) (6953 patients)	Not analyzed for glucocorticoids	Hydroxychloroquine, Remdesivir, and Lopinavir / Ritonavir can decrease the duration of symptoms.	No	Not analyzed for glucocorticoids	Very low certainty of evidence. For steroids, the direct estimate was more credible than that in the network, given the high heterogeneity.	Glucocorticoids probably reduce mortality and the need of CMV in COVID-19 patients compared with the usual standard of care.

One study showed a higher proportion of use in survivors (48% vs 23%, *p* < 005)	Not explored	1 study reduced mortality by 62% once an ARDS was established OR 0.38 (95% CI = 0.20–0.72; p = 0.003)	Not explored	Not reported	Yes	Longer virus detection time in those who receive corticosteroid 15 vs 8 days *p* = 0.013	High heterogeneity in the analysis It focuses on the results of the RECOVERY group already analyzed.	Observational studies do not allow inference of a definite beneficial effect of glucocorticoids; RECOVERY shows a favorable result, but more studies are required.

Not explored	Not explored	Only recommended for severe cytokine storm disease	Not explored	Not reported	Yes	Increase the virus clearance time	It does not specify the method of selection and evaluation of the quality of the articles; rather, it focuses on multiple drugs.	They consider the possibility of combined management between immunomodulators and antivirals.

It generally reduces mortality RR: 0.83, 95% CI = 0.75–0.93. p < 0.001. Reduces the requirement of CMV RR = 0.64 (95% CI = 0.51–0.81)	Mild to moderate reduction of 12 vs 13 days	Reduction in the requirement of CMV RR = 0.77 (95% CI = 0.62–0.95)	Not analyzed	Highest possibility of discharge on day 28	No	No	Preliminary report Patients with CMV were younger at the time of randomization.	The use of dexamethasone is associated with lower mortality at day 28 among patients with COVID-19 who require in-hospital management.

Not explored	Not explored	It is raised as a possibility of salvage therapy for critically ill patients.	Not explored	Not reported	No	No	Literature review focused on the management guidelines and pathophysiology of COVID-19	The possibility of using glucocorticoids is raised based on the possible immunomodulatory effects and pathophysiology of COVID-19 despite the lack of evidence of benefit in the face of the public health emergency.

41.8% reduction in mortality (13.9% vs 23.9%, OR = 0.51 [95% CI = 0.27–0.96], *p* = 0.044) – NNT 10)	Not explored	Lower mortality in ARDS 26.2 vs 60%, OR = 0.23 [05% CI = 0.08–0.71], *p* = 0.014	Not specified after treatment	Not reported	No	No	Higher proportion of patients with hematologic disease, presence of confusion and less use of other immunomodulators (hydroxychloroquine and tocilizumab) in the control group; lower levels of inflammatory markers. Less use of steroids in patients with diabetes.	Lower mortality in steroid-treated patients; no differences in mortality between usual doses and steroid pulses

65% mortality reduction OR: 0.35 (95% CI: 0.19–0.65)	Reduction in hospitalization time OR: -6.65 (95% CI: -10.93 to -2.37)	71% reduction in CMV requirement OR: 0.29 (95% CI = 0.14–0.65)	Not explored	79% greater probability of correcting the SLC at day 7 of follow-up (OR = 1.8; 95% CI = 1.2–2.7)	Yes	No differences between the groups regarding superinfection, kidney injury, HVD, or Cx events	Higher proportion of diabetic patients in the control group. Greater use of NIMV in the treatment group; all patients in the treatment group received some type of anticoagulation.	In COVID-19 patients with SLC, the use of methylprednisolone, with or without Tocilizumab, can accelerate respiratory recovery and decrease mortality and the need for CMV.

No difference in healing 73 (84.9%) 1 < vs 5 (83.3%) *p* = 0.85 or discharge 13 (15.1%) vs 3 (16.7%)	No differences at discharge.	Not described	No differences in the duration of fever *p* = 0.19 and the time of return to a normal temperature *p* = 0.68	No differences in the duration of fever No differences in the resolution time of the pulmonary findings by CT 11 (9-14) vs 11 (8-15) p = 0.87	Yes	No differences between the presence of hypokalemia and hyperglycemia	The steroid treatment group had worse risk markers, especially lymphopenia and elevated CRP.	Glucocorticoid therapy did not significantly influence the clinical course, the presence of adverse events, or the resolution of pneumonia in COVID-19 patients. More studies are required to establish the utility, dose, and time of initiation of glucocorticoid treatment.

Decrease in mortality combined with Tocilizumab (OR = 0.02, 95% CI = 0.0004–0.835, *p* = 0.04	Not explored	Need for CMV OR: 0.28 (95% CI = 0.019–4.19) *p* = 0.356	Not explored	Marked decrease in CRP	No	No	Limited to exclusive management of SLC; they pose less progression to death and OTI with the use of steroids but this is not reflected in the results.	The early use of GC pulses can control CRS, with a lower requirement for the use of tocilizumab and a decrease in events such as intubation and death.

108/136 (79.4%) patients receive methylprednisolone with greater use in critically ill patients where the doses were also higher. In the discussion, a controlled use is proposed given the risk of immunosuppression adjusted to ra+L25+J15:R16								

A deleterious effect is evidenced in patients receiving steroids with increased mortality in severely ill patients 59.8% vs 39.7%, p = 0.0005 and greater deterioration (progression) of the disease. Without disease progression 144 (17.7%) vs progression in severity 107 (47.4%) < 0.0001								Administration of oseltamivir or ganciclovir could be beneficial in reducing mortality in critically ill patients.

No one died	Discharge of 71.42% of patients Average stay 7.8 days	No patient required escalation in the management of the disease or presented deterioration.	77.98% reduction in peak CRP levels (129.52 ± 72.05) to 40.73 ± 49.28	Not reported	Yes	1 Patient with hyperglycemia	Observational study, case series type with exclusive in-hospital follow-up.	A short pulse of steroids is well tolerated and can mitigate proinflammatory states and improve patient outcomes.

Mortality	Hospital stay	Severity Progression	Signs and Symptoms	Other Findings	Reported	Explanation		

No reduction in early ARDS ( < 14 days) OR 0.57 (95% CI: 0.25-1.32) with NNT of 10 rel 818 harm / 5 benefit. No reduction in late ARDS (>14 days) OR: 0.58 (95% CI 0.22–1.53) with NNT of 15 rel 6 harm/21 benefit.	Not explored	Not explored	—	Not explored	No	No	High heterogeneity for early ARDS I2 test (52.7%, 95% CI = 0–85.1%) - X 2-test *p* = 0.12).	Current evidence does not support the use of corticosteroids in the treatment of ARDS in both early and late stages.

No reduction OR: 0.77 (95% CI 0.58–1.03, *p* = 0.079) In 6 studies (weighing less than 5%) there was a reduction in the risk of death OR: 0.25 (95% CI 0.13–0.48) In the others OR: 1.00 (95% CI 0.73–1.37)	Not explored	Not explored	—	Not explored	Yes	Two studies show an increased risk of lung infection OR: 2.24 (95% CI 1.21–4.13, *p* = 0.01) (I2 = 7, *p* = 0.30)	High heterogeneity between studies. (I2 = 70%, p < 0.001)	Based on existing studies, the favorable impact of the use of corticosteroids on mortality in patients with ARDS cannot be confirmed.

By day 28: General hospital mortality 20 vs. 33% (*p* = 0.006); Those who received early management (36 vs. 49%; risk ratio 0.76, 95% CI 0.59–0.98, I2 = 17%, *p* = 0.035) With the addition of ACEs hydrocortisone reduction in mortality 37 (20%) 45 (33%) OR: 0.48 (0.29–0.81), *p* = 0.006.	Shorter time to reach extubation, OR: 2.59 (95% CI 1.95–3.43); *p* = 0.001. Fewer days in ICU: (10.8 ± 0.71 vs. 6.4 ± 0.85; *p* = 0.001)	Day 28: Fewer patients die before achieving extubation (12 vs. 29%; *p* = 0.001) Day 28: Greater number of extubated patients 80 vs. 50%; *p* = 0.001	—	Reduction in CRP and interleukin levels in those who received glucocorticoid	Yes	No increase in nosocomial infection in the treatment group. There is an increase in return to CMV at day 28 (24 (16%) vs 4 (6%) OR: 4.04 (1.31–12.43), *p* = 0.015) without SOFA and age adjustments.	Publication bias was not ruled out. Low proportion of ACE's events and absence of baseline data from individuals prior to the start of methylprednisolone	The use of methylprednisolone accelerates the improvement of ARDS, reducing in-hospital mortality.

Possible reduction at 90 days in all-cause mortality in 86 out of 1000 patients (with up to 161 fewer or 19 more deaths). RR: 0.77; 95% CI 0.57–1.05; I2 = 27%; low-certainty evidence. No difference at 180 days RR: 0.99, 95% CI 0.64–1.52	Four studies evaluated 28-day follow-up without the need for a ventilator in 494 patients. The CMV-free time increased 4.09 days, 95% CI: 1.74–6.44, I2 = 36%; low-certainty evidence.	4 studies (368 patients) found a median decrease in CMV time of 5 (IQR 3–8) vs median of 9.5 (IQR 6–9.5 days): *p* = 0.002. Others of greater weight failed to show a reduction MD ?4.30 days; 95% CI: − 9.72–1.12; I2 = 93%; 277 patients; very low-certainty evidence.	—	Not explored	No	No	Substantial statistical heterogeneity was found for the estimates of effects that are explained by methodological or clinical differences between the studies.	There is insufficient evidence to determine with certainty whether any drug was effective in reducing mortality or decreasing CMV time in patients with ARDS. It was found that steroids could increase the number of days without mechanical ventilation. Most of the findings are of low or very low certainty; therefore, there is little confidence in the findings.
